# Efficacy of pain management for cattle castration: A systematic review and meta-analysis

**DOI:** 10.1017/awf.2025.10016

**Published:** 2025-06-24

**Authors:** Emeline Nogues, Jane Stojkov, Biljana Jonoska Stojkova, Marina A.G. von Keyserlingk, Daniel M. Weary

**Affiliations:** 1 Animal Welfare Program, Faculty of Land and Food Systems, The University of British Columbia, 2357 Main Mall, Vancouver, BC, V6T 1Z4, Canada; 2 Applied Statistics and Data Science Group, Department of Statistics, The University of British Columbia, 3178-2207 Main Mall, Vancouver, BC, V6T 1Z4, Canada

**Keywords:** Analgesia, animal welfare, local block, NSAID, orchiectomy, pain

## Abstract

Much research has assessed methods of pain control for cattle castration, but there remains a lack of consensus regarding best practice. We conducted a systematic review and meta-analysis of published research including both an untreated control (i.e. castrated without pain mitigation) and at least one unimodal or multimodal analgesia treatment (i.e. castrated with a local anaesthetic alone, or in combination with a non-steroidal anti-inflammatory drug) to summarise findings on castration pain management. Studies were included if they castrated by surgery, elastration or crushing, and reported at least one of the following outcomes: cortisol, change in bodyweight, foot stomping, wound licking, a subjective assessment of pain using a visual analogue scale, or stride length. Our search identified 383 publications, of which 17 were eligible for inclusion. Most publications focused on surgical castration (n = 14), and the most frequently reported outcome was blood cortisol (n = 13). None of the included studies were assessed as having a low risk of bias, mostly due to a lack of reporting blinding procedures and reasons for missing data. Using a three-level random effect model, we concluded that multimodal analgesia reduced blood cortisol concentrations in the first hour following surgical castration in comparison to the control group; this effect was diminished but still evident at 3 and 4 h, but not beyond at 6, 12 and 24 h. Too few data were available to meaningfully assess other outcomes and methods. Variability in methods and outcomes between studies, and risks of bias, hinder our capacity to provide science-based recommendations for best practice.

## Introduction

Male calves reared for meat are often castrated, regardless of whether they were born on beef or dairy farms (Coetzee *et al.* 2010). Castration is performed to reduce aggressiveness and unwanted sexual behaviour, as well as improve the texture and flavour of the meat (Seideman *et al.* 1982). The three most common castration methods are: (1) surgical (i.e. orchiectomies) consisting of an incision of the scrotum followed by removal of the testes by pulling, cutting or twisting of the spermatic cord; (2) elastration, using rubber ring or band to apply continuous pressure at the scrotal neck and thus interrupt blood flow until the tissues become necrotic and eventually slough off; and (3) crushing the scrotal neck (e.g. with the Burdizzo clamp), again interrupting blood flow to the testes and other tissues distal to the injury (Stafford *et al.* 2000).

Castration is recognised as causing pain in cattle by both producers and veterinarians (Edwards-Callaway *et al.* 2023), regardless of the choice of method. Pain due to this procedure has been the focus of much research, but between-studies variability has made it difficult to draw conclusions regarding which methods and analgesic treatments cause the least pain. Differences in the age at which animals are castrated can affect the experience and expression of pain (Meléndez *et al.* 2017), and a wide range of ages have previously been assessed: from a few days after birth (e.g. Boesch *et al.* 2008) to over six months old (e.g. Dockweiler *et al.* 2013). Many different clinical, physiological and behavioural outcomes have also been used to assess castration pain (for a review, see Coetzee 2011). Although the majority of studies to date have focused on acute pain (e.g. Meléndez *et al.* 2018a), some work also considers longer time-frames (e.g. Marti *et al.* 2017), but disparities in the choice of time-points for data collection makes comparisons difficult.

Minimising negative affective experiences, such as pain, is a key component for improving animal welfare (Fraser *et al.* 1997). To relieve the pain caused by castration, cattle can be provided local anaesthetics (intra-testicular and subcutaneous in and around the scrotum) and systemic analgesia (most frequently with non-steroidal anti-inflammatory drugs [NSAIDs]). Currently, practices vary among practitioners (Johnstone *et al.* 2021), countries and industries, as well as by castration method and age at the time of castration. For example, in Canada, the dairy industry requires the use of both local anaesthesia and systemic analgesia regardless of age and method (National Farm Animal Care Council 2023), while the beef industry only requires pain control for bulls older than six months of age (National Farm Animal Care Council 2013). The American Veterinary Medical Association suggests using local anaesthesia and systemic analgesia for surgical and band castration (AVMA 2024). A more comprehensive understanding of post-operative pain due to castration could help inform recommendations.

Studies on the efficacy of multimodal analgesia (e.g. using a combination of local anaesthetic and NSAID) and unimodal analgesia (e.g. local anaesthetic alone) sometimes report conflicting results, as noted by Canozzi *et al.* (2017) in their systematic review. These authors found no consistent effect of castration of beef cattle on cortisol (up to 2 h after the procedure) or on average daily gain (ADG), regardless of the castration method and pain mitigation strategy used. However, this review did not include studies that used a combination of local anaesthetic and NSAID, and did not include studies on dairy breeds. Those cases have been included in the current review to address this knowledge gap. Canozzi *et al.* (2017) focused on cortisol, vocalisations, and weight gain, but as mentioned above, many other pain-related outcome measures have been investigated in the context of castration. We focused on those most frequently reported in the literature: plasma and salivary cortisol, ADG, pain behaviours (i.e. foot stomping, wound licking), a subjective pain rating using a visual analogue scale (VAS), and stride length.

This systematic review aimed to summarise current findings in the literature regarding post-operative pain following castration in cattle (both beef and dairy), first by evaluating the efficacy of multimodal analgesia (local anaesthesia and NSAID) in mitigating pain compared to cattle castrated without any pain medication, and second by exploring the efficacy of local anaesthetic alone or compared to multimodal analgesia.

## Materials and methods

### Protocol and registration

The protocol for this systematic review was prepared following PRISMA-P guidelines (*Page et al.* 2015). This protocol was deposited on June 18^th^, 2020 in The University of British Columbia’s digital repository (cIRcle) available at https://doi.org/10.14288/1.0391907, and registered with the Systematic Reviews for Animals and Food (SYREAF), available at https://syreaf.org/protocols/.

### Eligibility criteria

#### Study design and population

Experimental intervention studies were eligible for inclusion if they involved bovine calves and young stock that underwent only castration, in both randomised and non-randomised trials. Observational studies or studies with another concurrent painful procedure (e.g. disbudding) were not eligible for inclusion.

#### Intervention and comparator group

Studies were eligible if they provided a within method comparison including an untreated control group (for which pain was not mitigated), and a treated control group (either with local anaesthetic alone, or combination of local anaesthetic and an NSAID).

#### Outcome measures

Studies were eligible if they reported at least one of the following outcome measures: plasma cortisol; saliva cortisol; ADG; two behavioural measures thought to relate to pain (i.e. foot stamping and wound licking); a subjective pain rating recorded using a VAS, or stride length. These outcome measures were determined *a priori* by EN and JS. Cortisol measures had to be reported at least once within the first 24 h following the castration procedure. ADG, pain behaviours, VAS and stride length had to be reported at least once within the first six weeks after the procedure.

#### Language and type of publication

Only literature published in English was eligible, with no restriction on publication date. Both peer-reviewed articles and grey literature reports (such as conference proceedings and theses) were eligible but were required to: (1) be publicly available; (2) provide detailed explanation of the experimental design; and (3) provide measures of variation (e.g. SD). If the same study was described in both the grey literature and in a peer-reviewed journal article only the latter was used.

### Information sources

Electronic searches of relevant published literature were conducted on the Web of Science (https://apps.webofknowledge.com), the Agricultural & Environmental Science Database (https://search-proquest-com.ezproxy.library.ubc.ca/agricenvironm/), and Medline (Ovid; http://ovidsp.dc2.ovid.com). Additionally, unpublished literature such as doctoral and master theses were searched using the ProQuest Dissertations and Thesis Database (https://search.proquest.com/databases/).

### Search strategy

The research team consulted with a librarian from The University of British Columbia to develop the following search parameters, then applied this to all databases: (calf OR calves OR bull OR bulls OR cattle OR bovine*) AND (castrat* OR orchiectom* OR gonadectom* OR burdizzo) AND (anesthetic* OR *caine OR NSAID OR metacam OR meloxicam OR flunixin OR banamine OR ketoprofen OR anafen OR non-steroidal* OR anti-inflammator* OR analgesi* OR “pain control*” OR “pain mitigation*” OR “pain strateg*” OR “control* pain” OR phenylbutasone OR carprofen OR “salicylic acid” OR aspirin).

### Study selection

Three searches on each of the three databases were completed on the following dates: June 24^th^ 2020, March 13^th^ 2023, and, lastly, July 17^th^ 2024. The results from all searches and from all databases were pooled and downloaded to the bibliographical management software EndNote (Philadelphia, PA, USA). Records from EndNote were then uploaded into the systematic review software Covidence (Melbourne, VIC, Australia). Duplicates were removed prior to starting the screening process. Two reviewers (EN and JS) independently followed a two-step screening process following the eligibility criteria described above and using the Covidence software tools. At each step, the screening process was piloted by the reviewers using a subset of 20 studies.

#### Title and abstract screening

The first screening step assessed eligibility based on the information available in the title and abstract. Reviewers assessed the eligibility of the study by considering the following criteria: (1) use of the English language; (2) description of an intervention study related to castration in cattle; (3) reference to the use of pain mitigation (i.e. local anaesthetic and/or NSAID); and (4) mention of at least one of the outcome measures of interest (i.e. plasma cortisol, salivary cortisol, ADG, pain-related behaviours, VAS, or stride length).

To the question “*Based on the title and abstract, is this study eligible?*”, reviewers could answer ‘yes’, ‘maybe’, or ‘no’. Studies were excluded (i.e. no further screening) if both reviewers answered ‘no’. Studies were moved to the next screening step if both reviewers answered ‘yes’ or ‘maybe’. Any disagreement was discussed by the two reviewers and resolved by consensus.

#### Full-text screening

During the second screening step, the remaining studies were assessed for eligibility based on the full text. The following criteria were applied: (1) use of the English language; (2) description of an intervention study related to castration in cattle in isolation from any other painful procedure; (3) inclusion of a control group castrated with no pain mitigation, and at least one castrated treatment group (local anaesthetic alone, or local anaesthetic and NSAID in combination); and (4) reporting of at least one of the outcome measures of interest (either within the first 24 h or first six weeks following castration, according to the description provided in the outcome measure eligibility criteria section).

To the question “*Based on the full text, is this study eligible?*”, if both reviewers answered ‘yes’, then the study was included in the systematic review. If reviewers answered ‘no’, the reason for exclusion was recorded. Any disagreement on the eligibility of a study or on the reason for its exclusion was discussed by the two reviewers and resolved by consensus. If there were multiple reasons for excluding the study, the one listed was decided based on the following order: language, population, intervention, comparator, outcome.

### Risk of bias assessment

A risk of bias assessment was performed for each outcome measure using the revised Cochrane risk-of-bias tool for randomised trials (RoB 2; Chandler *et al.* 2016). The signalling questions outlined in this tool were modified as necessary (our version is available at https://doi.org/10.5683/SP3/AVESUR). Modifications aimed to adjust the tool for animal studies (e.g. by removing the question on participant’s awareness of the treatment they received) and to clarify understanding for the reviewers. Two reviewers completed the assessment independently, after pilot testing on four outcomes from two different studies. Disagreements between the reviewers were resolved by consensus.

### Data extraction

Data were extracted for meta-analysis from 15 of the 17 eligible studies; format of the data in the other two publications did not allow for extraction. Study-level data included first author last name, year of publication, castration method, age of the animals at the time of castration, and sample size for each treatment group and outcome measure. For treatment groups with pain mitigation, the data also included name of the drug (for both local anaesthetic and NSAID, as applicable), dose (in mL or mg kg^–1^), administration route (SC, IV, IM, intra-testicular, or oral) and timing relative to castration.

To extract data on the outcome measures of interest, authors were contacted by email, resulting in data from five studies being received. If the authors did not reply or no longer had access to the original dataset, data were extracted independently by two reviewers from the results section of the publication (ten studies). For data reported graphically (14 figures across ten studies), the same screenshot of the figure was used by both reviewers to extract data using the software WebPlotDigitizer (Rohatgi 2022). The x-values representing the time-points were corrected based on the information provided in the material and methods section of the publication. The differences in the y-values (value of the outcome measure at each time-point) collected by the two reviewers were calculated. If the calculated difference was less than 0.5, the average value of both reviewers was used in the meta-analysis. If the difference was greater than 0.5, the reviewers reviewed the data-point together and resolved the inconsistency by consensus before inclusion in the meta-analysis. The value of 0.5 was decided by consensus among the authors, as some minor inconsistencies were expected due to the extraction method.

If the cortisol concentrations were reported in ng mL^–1^, the values were converted to nmol L^–1^. One study graphically presented the cortisol concentrations as the relative change from the baseline (Stafford *et al.* 2002); the actual concentration was calculated using the baseline values provided in Table 1 of the publication.

**Table 1.** Characteristics of the 17 studies included in the systematic review of efficacy of pain management for cattle castration. Studies compared cattle when castrated without any pain medication versus with either multimodal (local anaesthetic [LA] and NSAID) or unimodal (local anaesthetic [LA] only) analgesia

Study	Breed	Age	Method(s)	Intervention group(s)	Outcome(s)	Data source
Ballou *et al.* (2013)	Holstein	3 months	Surgical	LA + NSAID	Plasma cortisol	Extracted
Boesch *et al.* (2008)	Holstein-Friesian Brown Swiss Beef × Dairy	2–7 days	Crushing	LA	Plasma cortisol Foot stamping Wound licking	Authors
Earley & Crowe (2002)	Friesian	5.5 months	Surgical	LA LA + NSAID	Plasma cortisol ADG	Extracted
Fisher *et al.* (1996)	Friesian	5.5 months	Surgical Crushing	LA	Plasma cortisol ADG	Extracted
Laurence *et al.* (2018)	Brahman	6–8 months	Surgical	LA LA + NSAID	Plasma cortisol ADG	Not available
Martin *et al.* (2022)	Holstein	16–20 weeks	Surgical	LA LA + NSAID	Serum cortisol Foot stamping Wound licking	Extracted
Meléndez *et al.* (2018b)	Angus crossbred	7–8 months	Surgical	LA LA + NSAID	Salivary cortisol ADG VAS Stride length Foot stamping Wound licking	Authors
Nordi *et al.* (2019)	Angus	6 months	Surgical Elastration	LA + NSAID	Salivary cortisol ADG VAS Stride length	Authors
Onda *et al.* (2021)	Holstein	5 months	Surgical	LA	Serum cortisol	Authors
Park *et al.* (2018)	Korean cattle	6 months	Surgical	LA + NSAID	Plasma cortisol ADG	Extracted
Stafford *et al.* (2002)	Friesian	2–4 months	Surgical Elastration (2) Crushing	LA LA + NSAID	Plasma cortisol ADG	Extracted
Stewart *et al.* (2010)	Friesian	4 months	Surgical	LA	Plasma cortisol	Authors
Sutherland *et al.* (2013)	Holstein	3 months	Surgical	LA + NSAID	Foot stamping	Extracted
Thüer *et al.* (2007)	Simmental Simm. × Red Holstein	21–28 days	Elastration Crushing	LA	Serum cortisol Foot stamping	Extracted
Ting *et al.* (2003)	Holstein-Friesian	13 months	Crushing	LA	Plasma cortisol ADG	Extracted
Vindevoghel *et al.* (2019)	Brahman	6–8 months	Surgical	LA + NSAID	Foot stamping Wound licking	Not available
Webster *et al.* (2013)	Holstein-Friesian	2–3 months	Surgical	LA LA + NSAID	Plasma cortisol ADG Foot stamping Wound licking	Extracted

Data source describes how the data was obtained for meta-analysis: Authors (authors shared their original dataset), Extracted (data was obtained from tables and/or graphs in the publication), Not available (format in the publication did not allow for extraction).

ADG: Average daily gain; VAS; Visual analogue scale.

### Meta-analysis

Meta-analysis was performed only on plasma cortisol concentrations after surgical castration, as this was the only reported outcome and method for which there was sufficient data with comparable time-points to carry out the analysis (at least three studies at each time-point). The extracted data are available at https://doi.org/10.5683/SP3/AVESUR. Analysis were conducted in R (version 4.2.3; R Core Team 2023), package *meta* (version v; Balduzzi *et al.* 2019).

Plasma cortisol measurements collected from the first 24 h after castration were used in the meta-analysis. Time-points were constructed at 1, 3, 4, 6, 12 and 24 h. If measurements had been taken 15 min before or after one of the target time-points listed above, these were included and classified to the closest hour. Different studies contributed measurements at different target time-points, introducing direct comparability issues.

One of the studies (Stafford *et al.* 2002) contributed two data-points, each corresponding to different sub-methods (within surgical and elastration methods), with each sub-method being applied to independent test animals. This creates dependencies in the observations within a single time-point and within castration method, resulting in additional level of within-study heterogeneity in addition to the already existing between-study heterogeneity.

Statistical analyses were conducted using a three-level random effect model fitted at each available time-point for each of the castration methods to analyse the effects of multimodal analgesia (local anaesthetic and NSAID) to the control group with no pain medication on plasma cortisol concentration (Fernández-Castilla *et al.* 2020). Random intercept effects were specified for study and group, which allowed estimation of between- and within-study heterogeneity. The main estimate of interest is the overall (studies) mean cortisol plasma difference between the multimodal analgesia and control groups. The overall (studies) and within-study group estimates were all obtained as mean differences between the groups, so the effect size would be interpretable on the plasma cortisol measurement scale (nmol L^–1^; Luo *et al.* 2018).

We used a three-level model with random effects for study and group, which allowed us to obtain between- and within-study heterogeneity (τ^2^). We used the restricted maximum likelihood estimator (Viechtbauer 2005) for estimating the between- and within-study heterogeneity τ^2^. Confidence intervals for both were obtained using the profile-likelihood method. We applied the inverse variance method for estimating the percentage of weight for each study. Pooled estimates in each analysis were obtained as unstandardised mean difference (as a weighted average of the study level effect sizes; Harrer *et al.* 2021), which was chosen for improved interpretability on the plasma cortisol original scale. I^2^, which represents the proportion of variation across studies that is due to heterogeneity rather than small sample size or by chance (Higgins *et al.* 2003), was also obtained from our models. We report I^2^, since it is an intuitive measure of the inconsistency of study results.

In five studies, variation was reported using the standard errors. For consistency between studies, we calculated the standard deviations by multiplying the standard errors with the square root of the sample size (Thalheimer & Cook 2002; Shi *et al.* 2020).

Similarly, the secondary statistical analysis (local anaesthesia only compared to the control group) was conducted by fitting a three-level random effect model specified in similar fashion as the model in primary statistical analysis described above. Plasma cortisol was specified as the response variable, group as fixed effect (two levels: local anaesthetic and control groups) and random intercepts for both study and group.

In this analysis, we could only estimate the effectiveness of multimodal (local anaesthetic and NSAID) or unimodal (local anaesthetic only) analgesia treatments by comparing those to the control group with no pain medication. The overall mean plasma cortisol difference between the multimodal analgesia treatment (local anaesthetic and NSAID) and the null control was obtained only for time-points that contained at least three observations.

## Results

### Study selection and characteristics

**Figure 1.**
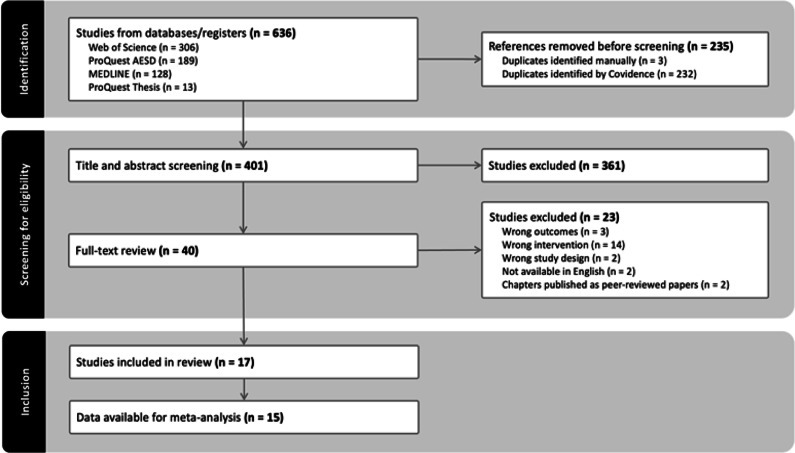
PRISMA (Preferred Reporting Items for Systematic Reviews and Meta-Analyses) study flow diagram (adapted from PRISMA 2020; Page *et al.* 2021) used for the systematic of efficacy of pain management for cattle castration based on three searches conducted in June 2020, March 2023, and July 2024.

Our search yielded 383 unique publications, 17 of which met the eligibility criteria and were included in our review (Figure 1); all were peer-reviewed journal articles. Key characteristics (breed and age of the animals, castration methods, intervention groups, outcomes, and data source) of the eligible studies are reported in Table 1, and the respective analgesia protocols (drugs, route, dose and timing of administration) are presented in Table 2. Data from two studies could not be extracted: Laurence *et al.* (2018) because data were presented as deviation from the non-castrated control group, and Vindevoghel *et al.* (2019) because the frequencies of behaviours were summed and presented as a score.

**Table 2.** Local anaesthetics and NSAID protocols applied in the 17 studies included in the systematic review of efficacy of pain management for cattle castration

Study	Local anaesthetic	Dose and route	Timing	NSAID	Dose and route	Timing
Ballou *et al.* (2013)	Lidocaine	12 mL IT + SC	Imm. prior	Flunixin	2 mg kg^–1^ IM	Imm. prior
Boesch *et al.* (2008)	Lidocaine OR Bupivacaine	10 mL IT + SC	20 min prior	N/A	N/A	N/A
Earley & Crowe (2002)	Lidocaine	18 mL IT + SC	20 min prior	Ketoprofen	3 mg kg^–1^ IV	20 min prior
Fisher *et al.* (1996)	Lidocaine	22 mL IT + SC	15 min prior	N/A	N/A	N/A
Laurence *et al.* (2018)	Lidocaine	2mg kg^–1^ IT + SC	5 min prior	Meloxicam	0.5 mg kg^–1^ SC	30 min prior OR Imm. after
Martin *et al.* (2022)	Lidocaine OR Bupivacaine	8–10 mL IT + SC	10 min prior	Meloxicam	1 mg kg^–1^ oral	10 min prior
Meléndez *et al.* (2018b)	Lidocaine	30 mL IT + SC	30 min prior	Meloxicam	0.5 mg kg^–1^ SC	30 min prior
Nordi *et al.* (2019)	Lidocaine	30 mL IT + SC	30 min prior	Flunixin	2.2 mg kg^–1^ SC	30 min prior
Onda *et al.* (2021)	Lidocaine	10 mL IT + SC	20 min prior	N/A	N/A	N/A
Park *et al.* (2018)	Lidocaine	12 mL SC	Imm. prior	Flunixin	2 mg kg^–1^ IV	Imm. prior
Stafford *et al.* (2002)	Lidocaine	10 mL IT + SC	20 min prior	Ketoprofen	3 mg kg^–1^ IV	20 min prior
Stewart *et al.* (2010)	Lidocaine	17 mL IT + SC	10 min prior	N/A	N/A	N/A
Sutherland *et al.* (2013)	Lidocaine	12 mL IT + SC	Imm. prior	Flunixin	2 mg kg^–1^ IM	Imm. prior
Thüer *et al.* (2007)	Lidocaine	10 mL IT + SC	5 min prior	N/A	N/A	N/A
Ting *et al.* (2003)	Lidocaine	22 mL IT + SC	20 min prior	N/A	N/A	N/A
Vindevoghel *et al.* (2019)	Lidocaine	2 mg kg^–1^ IT	5 min prior	Meloxicam	0.5 mg kg^–1^ SC	Imm. after
Webster *et al.* (2013)	Lidocaine	25 mL IT + SC	20 min prior	Flunixin	1.1 mg kg^–1^ IV	20 min prior

IT = intratesticular; SC = subcutaneous; IM = intramuscular; IV = intravenous; Imm. = immediately.

In 14 of the 17 eligible studies, bulls were castrated surgically, five by crushing, and three by elastration (some included multiple methods: Fisher *et al.* 1996; Stafford *et al.* 2002; Thüer *et al.* 2007; Nordi *et al.* 2019). One of the studies included two different elastration treatments and therefore was duplicated in our data-set (some bulls were castrated with two rubber rings, some bulls with a band; Stafford *et al.* 2002). Six of the 17 eligible studies included both multimodal and unimodal analgesia as interventions, five included only multimodal analgesia, and six studies only considered unimodal analgesia (i.e. local anaesthetic only). Lidocaine was the local anaesthetic of choice in all 17 studies, but two studies had a second treatment group with bupivacaine as the local anaesthetic (Boesch *et al.* 2008; Martin *et al.* 2022). Of the eleven studies that provided an NSAID, five used flunixin meglumine, four meloxicam, and two ketoprofen. Studies often reported multiple outcome measures: 13 studies measured blood cortisol (ten from plasma and three from serum samples), nine measured ADG, seven foot stomping, five wound licking, two salivary cortisol, two stride length, and two used a VAS pain score.

### Risk of bias assessment

The risk of bias assessment was conducted on each outcome for all eligible studies (detailed assessment is available at https://doi.org/10.5683/SP3/AVESUR). No study was assessed as ‘low risk’. For three studies, all outcomes were assessed as ‘some concerns’ (Ting *et al.* 2003; Stewart *et al.* 2010; Meléndez *et al.* 2018b), as well as a single outcome in a fourth study (Park *et al.* 2018). All other outcomes and studies had a high-risk. These ratings reflect the lack of information available to answer at least one signalling question in all studies and outcomes.

All studies and outcomes were also assessed as either ‘some risk’ or ‘high risk’ of bias in the second domain, pertaining to bias due to deviations from intended interventions. Only four studies made clear statements regarding deviations from intended interventions (e.g. additional drugs provided during the study period), including one study explicitly stating that deviations occurred and that these deviations were unbalanced between treatments and likely to have affected the outcome (Nordi *et al.* 2019). The other three either failed to provide information or clearly stated that there was no blinding of personnel at the time of the intervention with regards to the analgesia treatment provided (Fisher *et al.* 1996; Webster *et al.* 2013; Martin *et al.* 2022).

### Meta-analysis

**Figure 2.**
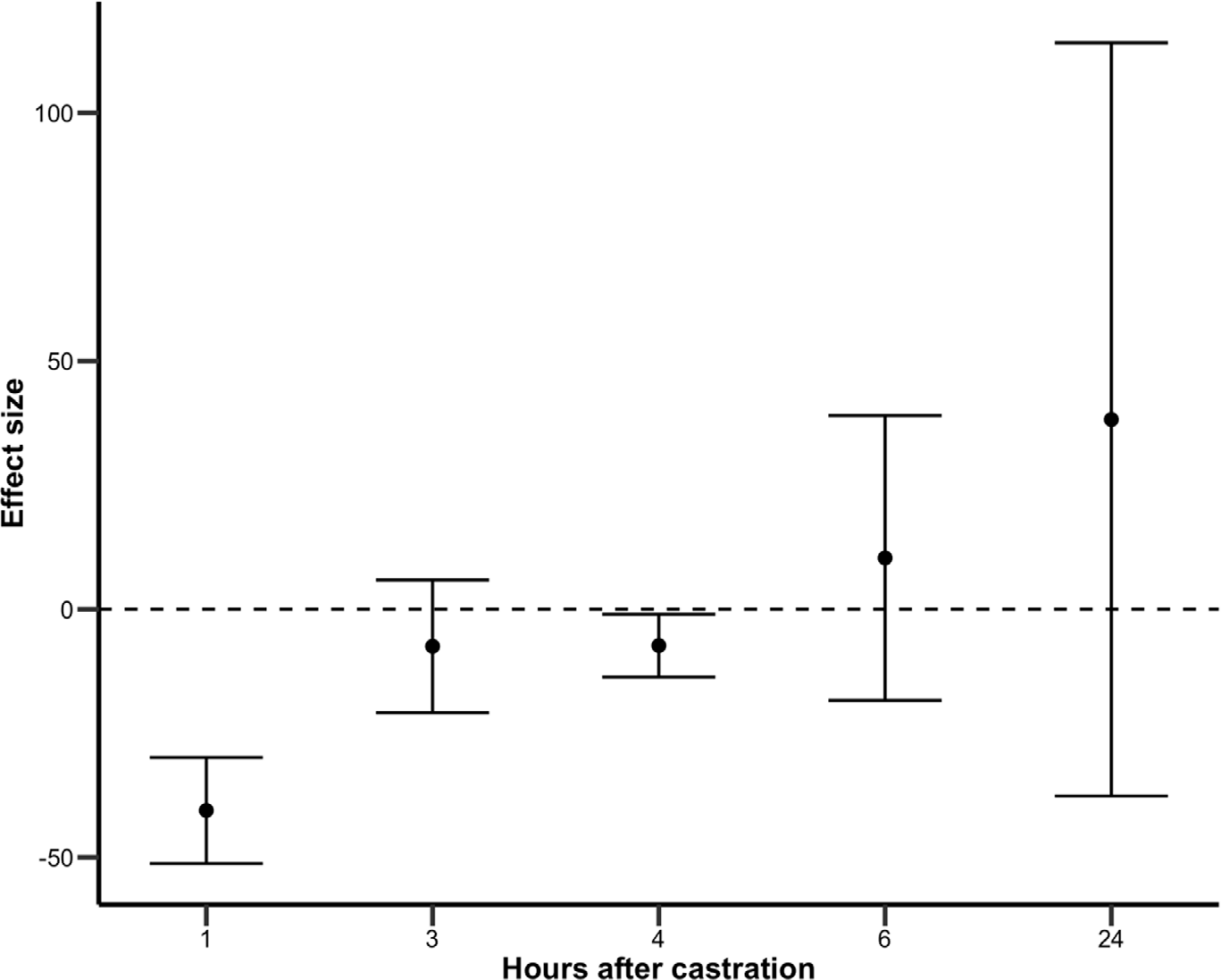
Estimated effect of multimodal analgesia on blood cortisol (in nmol L^–1^) compared to control animals (not provided any analgesia) during the first 24 h after surgical castration. Results at each time-point are based on the meta-analysis of data extracted from 2, 2, 4, 5 and 3 studies, respectively.

Sufficient data were available to obtain overall group estimates only for blood cortisol concentrations following surgical castration with or without multimodal analgesia (Figure 2). These data originated from five different studies (Stafford *et al.* 2002; Ballou *et al.* 2013; Sutherland *et al.* 2013; Webster *et al.* 2013; Park *et al.* 2018); it is to be noted that all were assessed as having a high risk of bias for this outcome.

In comparison to the control group, multimodal analgesia was most effective in reducing blood cortisol concentration in the first hour following castration; continued to provide some benefit 3 and 4 h after castration, but not at subsequent time-points (6 and 24 h after castration; Table 3).

**Table 3.** Estimated effect of the LA+NSAID relative to the control group not provided analgesia on plasma cortisol concentration (expressed in nmol L^–1^) at each time-point (expressed in hours after castration) for the surgical method. Results at each time point are based on the meta-analysis of data extracted from nStudies, respectively. Effect sizes, three heterogeneity estimates (between- and within-study tau estimates, and I^2^) and their 95 percent confidence intervals are presented

Hours	nStudies	Effect [CI95%]	Between-study Tau [CI95%]	Within-study Tau [CI95%]	I^2^ [CI95%]
1	2	–40.8 [–51.4, –30.1]	0.03 [0, 3.2]	0.03 [0, 3.2]	0 [0, 0.896]
3	2	–7.6 [–20.9, 5.7]	6.0 [0, 60.4]	0 [0, 3.2]	0 [0, 0.896]
4	4	–7.4 [–13.7, –1.2]	0 [0, 3.2]	0 [0, 3.2]	0 [0, 0.792]
6	5	10.3 [–18.2, 38.8]	30.8 [0, 82.8]	3.6 [0, 35.7]	0.842 [0.673, 0.924]
24	3	38.1 [–37.7, 113.9]	66.5 [33.8, 210.4]	N/A	0.98 [0.964, 0.989]

The estimates for 1, 3 and 4 h after castration indicated low heterogeneity estimates, but high heterogeneity was found for the estimates at 6 and 24 h after castration (Table 3). Hence, the effect sizes estimated at 6 and 24 h were likely biased and should be interpreted with caution.

## Discussion

The primary objective of this review was to summarise findings in the literature regarding post-operative pain following castration in cattle, with or without multimodal analgesia (local anaesthetic and NSAID). Our secondary and tertiary objectives included a comparison of post-operative pain with or without local anaesthetic only, and the relative efficacy of these two approaches to analgesia. This work builds on Canozzi *et al.* (2017) who reviewed studies on pre-weaned beef calves published up until May 2015.

The main challenge in conducting this meta-analysis was the variability in methods used in the different studies. First, regarding castration method, we grouped techniques under three categories, but this omitted some of the variation within methods. Surgical castration could be performed by pulling (e.g. Nordi *et al.* 2019; Martin *et al.* 2022), cutting (e.g. Ballou *et al.* 2013; Sutherland *et al.* 2013), or twisting with a Henderson tool (Park *et al.* 2018; Webster *et al.* 2013), while elastration included the use of a single rubber ring, two rubber rings, or a band (e.g. Stafford *et al.* 2002). We did not distinguish between drugs and routes of administration but recognise that both factors likely contributed to the variability observed between studies. Third, apart from blood cortisol measures reported in 14 of the 17 studies (N.B. serum and plasma cortisol measures were grouped), the choice of outcome measures varied among studies. Fourth, despite most studies justifying time-points based on previous literature, the choice of the time-points at which outcomes were measured varied among studies. Finally, the age of the animals at the time of the procedure varied; among the 17 studies, age of castration ranged from two days to eight months. Thus, our study included multiple castration techniques, analgesia protocols and outcomes; we recognise this is a limitation of our work.

Following data extraction, we were able to obtain group estimates for the relative effect of providing local anaesthetic and NSAID on plasma cortisol concentration after surgical castration, in comparison with the control group. Sources of variability between studies as described above did not allow for other comparisons in the meta-analysis, thus also preventing us from addressing our secondary objectives for this review (i.e. comparing unimodal analgesia vs control, as well as unimodal vs multimodal). Our findings show that multimodal analgesia is effective, at least for the first few hours after castration. Canozzi *et al.* (2017) found no effect of the local anaesthetic alone 0.5 and 2 h after surgical castration. The effect we report here likely reflects the importance of adding an NSAID to a local block when seeking to reduce pain in the hours after castration.

The heterogeneity at the 6 and 24 h time-points contributes to the biases in the overall group estimates. This heterogeneity may be a consequence of including the Park *et al.* (2018) study, for which no data were available at previous time-points. The reported treatment means from this study were up to 20-fold higher than for other studies, potentially attributable to differences in method used to measure cortisol. The authors describe their use of a “*a cortisol salivary HS ELISA kit*” (Park *et al.* 2018; p 63), potentially rendering these data unreliable for blood.

Cortisol was used as a pain marker in most of the studies included in this review. This is comparable to findings from other systematic reviews, one on disbudding of cattle (Winder *et al.* 2018), and one on castration in piglets (Dzikamunhenga *et al.* 2014). In most cases, an increase in cortisol is recorded in response to the painful procedure, which can be mitigated with analgesia (Stafford & Mellor 2005), but cortisol responses lack specificity to pain, and should be seen more generally as an indicator of stress rather than pain *per se* (e.g. when isolating cattle from their peers: Boissy & Le Neindre 1997; when overstocking dairy cows: Fustini *et al.* 2017). The cortisol response also wanes before behavioural or immunological markers return to baseline (Adcock & Tucker 2018). For these reasons, the use of cortisol as a reliable marker of pain has been questioned (Ede *et al.* 2019), and interpretation of the findings of our review should be viewed with this in mind.

Regardless of outcome and study, important features of the methods were often unreported, contributing to the assessment as ‘some concerns’ in ten of the 46 outcomes assessed, and ‘high risk’ for all others. One area of concern is the lack of information on blinding of study personnel (including both caregivers providing the analgesia, and outcome assessors). This is particularly concerning for outcomes that involve some judgement such as behavioural outcomes or subjective ratings (e.g. using a VAS), as prior knowledge of the treatment might influence the assessment (Higgins *et al.* 2024). Similar concerns have been brought forward in previous systematic reviews on livestock (Sargeant *et al.* 2009), and more specifically on painful procedures in cattle (castration: Canozzi *et al.* 2017; disbudding: Winder *et al.* 2018). Reporting guidelines are available to limit the risk of biases, such as the REFLECT statement (reporting guidelines for randomised control trials in livestock and food safety; O’Connor *et al.* 2010; Sargeant *et al.* 2010). We encourage scholars to use this checklist when designing their study.

In this review, one inclusion criterion was that publications be in English. This criterion was established to ensure consistency in interpretation and analysis of the included studies. However, we acknowledge it constitutes a limitation as further relevant literature might be available in other languages.

### Animal welfare implications

Beyond the use of multimodal analgesia after surgical castration, our findings highlight the complexity of making evidence-based recommendations from the existing literature. Although this calls for further investigation, and for replication studies correcting the biases mentioned above, conducting studies on painful procedures without providing pain control raises ethical concerns. While the ultimate aim of such research may be to enhance welfare during these procedures, the lack of pain relief for the control group should be questioned. Alternative research methodologies that achieve similar goals without causing this harm should be prioritised. The results of the current study indicate that animals should be provided a local block and NSAID, providing a suitable baseline treatment for future work.

## Conclusion

Our meta-analysis shows that multimodal analgesia mitigates the increase in blood cortisol concentration in the first 4 h following surgical castration and that this effect wanes thereafter. Our review shows that many studies on post-operative pain management for cattle castration suffer from a lack of consistency, hindering our ability to make evidence-based recommendations. We encourage replication studies but discourage including test groups that are not provided pain control.

## Data Availability

The extracted data, risk of bias assessment tool and risk of bias assessment results are available at https://doi.org/10.5683/SP3/AVESUR.
